# Obtaining high-quality draft genomes from uncultured microbes by cleaning and co-assembly of single-cell amplified genomes

**DOI:** 10.1038/s41598-018-20384-3

**Published:** 2018-02-01

**Authors:** Masato Kogawa, Masahito Hosokawa, Yohei Nishikawa, Kazuki Mori, Haruko Takeyama

**Affiliations:** 1 Department of Life Science and Medical Bioscience, Waseda University, 2-2 Wakamatsu-cho, Shinjuku-ku, Tokyo 162–8480 Japan; 2 Computational Bio Big-Data Open Innovation Laboratory, AIST-Waseda University, 3-4-1 Okubo, Shinjuku-ku, Tokyo 169–0072 Japan; 3 Research Organization for Nano & Life Innovation, Waseda University, 513 Wasedatsurumaki-cho, Shinjuku-ku, Tokyo 162–0041 Japan; 4 PRESTO, Japan Science and Technology Agency (JST), 5-3 Yonban-cho, Chiyoda-ku, Tokyo 102-0075 Japan

## Abstract

Single-cell genomics is a straightforward approach to obtain genomes from uncultured microbes. However, sequence reads from a single-cell amplified genome (SAG) contain significant bias and chimeric sequences. Here, we describe Cleaning and Co-assembly of a Single-Cell Amplified Genome (ccSAG), a novel analytical workflow to obtain composite single-cell genomes with elimination of sequence errors. By the integration of ccSAG with a massively parallel single-cell genome amplification platform based on droplet microfluidics, we can generate multiple SAGs and effectively integrate them into the composite genomes quality equivalent to the data obtained from bulk DNA. We obtained two novel draft genomes from single gut microbial cells with high completeness (>96.6%) and extremely low contamination (<1.25%). Moreover, we revealed the presence of single nucleotide polymorphisms in the specific gene by sequence comparison at the single-cell level. Thus, the workflow yields near-complete genomes from uncultured microbes, and enables analyses of genetic heterogeneity within identical strains.

## Introduction

A large fraction of microbes cannot be cultured by traditional techniques. Thus, metagenomics, which does not require cultivation, has become a useful tool to understand microbial diversity. However, metagenomic data consist of fragmented and mixed sequences, so that predicted gene functions are difficult to link to specific organisms. As an alternative, single-cell genomics is now used to directly target specific microbial genomes and associated gene functions while avoiding the challenges of cultivating microorganisms or interpreting complex metagenomic data^1–3^.

In single-cell genomics, a single microbial cell is isolated, lysed, and whole-genome amplified, typically by multiple displacement amplification (MDA)^4^ using phi29 DNA polymerase and random primers. Although MDA generates sufficient quantities of replicated DNA with high fidelity and large fragment size, several issues may emerge. For example, MDA generally introduces chimeric artifacts by linking noncontiguous genomic regions. Genomic coverage is also severely biased, ultimately resulting in lack of coverage of some genomic stretches. In addition, contaminating DNAs are also amplified, and degrade the quality of the final sequence. Consequently, single-cell amplified genomes (SAG) obtained from uncultured microbes, especially those without reference genomes that can be used as control, may contain errors and are prone to misinterpretation. In most cases, such genomes are also fragmented and incomplete^5,6^.

To overcome these issues, various improvements have been introduced to experimental and computational methods. For instance, pico- or nanoliter reactions may suppress biased amplification during MDA^7–11^ and reduce contamination^12^. Indeed, we previously described MDA techniques^10,12^ that generate 10^5^ SAGs from picoliter droplets in four hours. On the other hand, several bioinformatics tools have been developed to identify and exclude contaminant and nontarget sequences^13–15^. For example, SPAdes was recently developed to assemble single-cell genomes despite nonuniform coverage and contamination with chimeras^16^. In addition, amplified genomes from multiple, closely related single cells, *e.g*., those with average nucleotide identity (ANI) 95% and likely belong to the same species, have been combined to overcome lack of genome coverage^6^,^17^. Single-cell genomes have also been assembled using metagenomic composite genomes as internal reference^18^. However, chimeric and contaminant fragments also accumulate when multiple single-cell or metagenomic data are combined, increasing the risk of misinterpretation and short contig production while improving genome completeness. Moreover, single nucleotide polymorphisms (SNPs) may disappear from the resulting composite genomes. Thus, these approaches may ultimately mask the innate characteristics of uncultured microbes, and obscure genetic and functional heterogeneity.

To address persistent issues of chimerism and improve SAG quality from environmental samples, we have developed Cleaning and Co-assembly of a Single-Cell Amplified Genome (ccSAG), a novel, systematic, and generalized workflow to remove potentially chimeric sequences and co-assemble multiple, closely related SAGs *de novo* into a near-complete genome. We then integrated this workflow into a massively parallel single-cell MDA platform based on microfluidic droplets^12^ to investigate microbes in the mouse gut. In addition, we assessed the performance of the method against jackknifing, a computational approach also designed to remove chimeras. We found that, in comparison to conventional tools, ccSAG generates composite single-cell genomes with overall quality equivalent to those assembled from bulk DNA. Importantly, coding sequences and gene clusters can be clearly inferred from such composite genomes. Moreover, we obtained two novel draft genomes from uncultured mouse gut microbes, in which SNPs have been preserved. Therefore, ccSAG provides, for the first time, the ability to link gene functions to uncultured microbes and to survey genetic heterogeneity in the same strain. We anticipate that ccSAG will advance single-cell microbiology in meaningful ways, and help illuminate the functional role of microbial dark matters.

## Results

### ccSAG workflow

**Figure 1:**
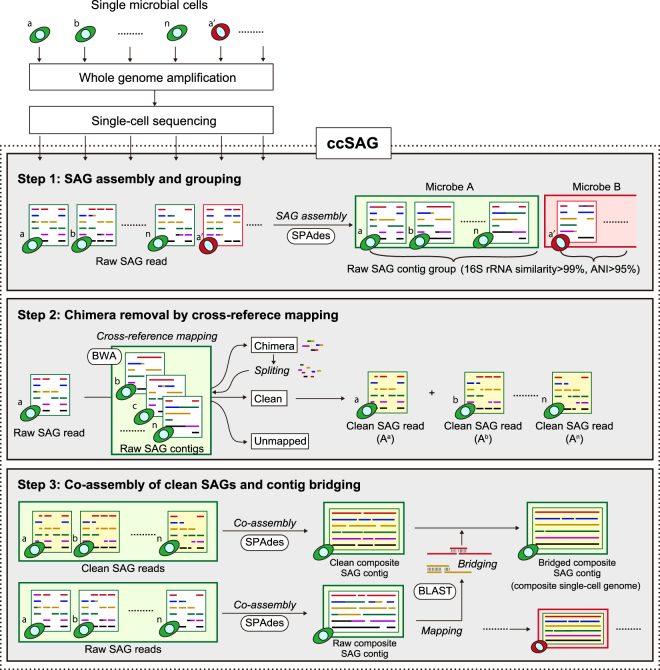
ccSAG workflow to clean and co-assemble SAGs into a composite single-cell genome. Single-cell whole genomes are first amplified from individual microbial cells, and processed to construct sequencing libraries. After multi-index single-cell sequencing, reads are assembled by SPAdes into raw SAG contigs, which are then grouped based on similarity and identity (step 1). Subsequently, SAG reads are iteratively mapped by BWA on other raw SAG contigs to identify and remove chimeras (step 2). Finally, clean SAG reads are co-assembled by SPAdes into clean composite SAG contigs, and bridged by BLAST using raw composite SAG contigs (step 3). The output is a clean, gap-free composite single-cell genome suitable for downstream analysis.

In ccSAG (Fig. 1), raw SAGs are first classified into groups based on 16S rRNA similarity ≥99% in the V3–V4 region and ANI > 95% in suitable sequences. After quality control of sequence reads, raw contigs are constructed from each SAG for use as cross-reference in the next step, in which each SAG read is mapped (see Methods) to multiple raw contigs in the same group, and classified as clean, potentially chimeric, or unmapped. Potential chimeras that partially align with raw contigs are split into aligned and unaligned fragments (>20 b) and remapped. Cycles of cross-reference mapping and chimera splitting are performed until chimeras are undetectable and fully unmapped reads are identified. In the final step, clean reads obtained from each SAG are co-assembled *de novo* as clean composite SAG contigs. Similarly, raw reads are co-assembled *de novo* as raw composite SAG contigs. By mapping the latter to the former, minor sequences represented in single SAGs only, but also align with clean contigs, are used to close gaps and generate bridged composite SAG contigs, which essentially comprise the composite single-cell genome for the SAG group.

### Chimera identification by cross-reference mapping

**Figure 2:**
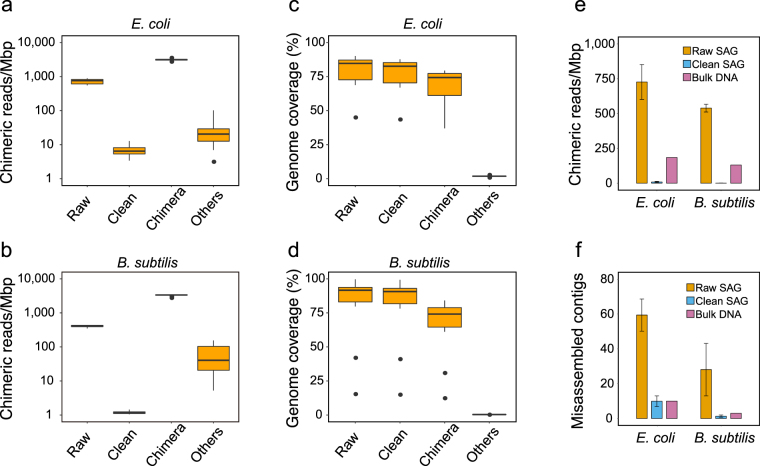
Quality of chimera identification and removal by cross-reference mapping and chimera splitting. All sequence reads from 12 each of *E. coli* and *B. subtilis* SAGs were classified as clean, potentially chimeric, and unmapped (others) in a single cycle of cross-reference mapping. (**a** and **b**) Chimeric reads and (**c** and **d**) genome coverage before and after cross-reference mapping. Boxes range from the 25th to the 75th percentile, with the centerline representing the 50th percentile. Outliers are shown as dots. (**e**) Chimeric reads per Mb and (**f**) misassembled contigs after *de novo* assembly of SAG reads cleaned by multiple cycles of cross-reference mapping and chimera splitting (mean ±S. d.). Error bars represent standard deviation. Data are from 12 SAGs before and after cleaning, and from 10 ng bulk genomic DNA equivalent to 2 × 10^6^ cells.

To evaluate the performance of ccSAG and optimize run parameters, we used 12 existing SAG data each for *E. coli* and *B. subtilis*, which have different GC % content and membrane structure^12^. Within each species, the SAGs were 100% similar at 16S rRNA fragments, had ANI > 98%, and thus were easily grouped in the first step. By cross-reference mapping to raw SAG contigs, reads were then classified as clean, potentially chimeric, and unmapped. Mapping to the *E. coli* and *B. subtilis* reference genome (Fig. 2a,b; Table 1) after one cycle indicated that reads classified as clean contained 7 ± 3 and 0.8 ± 0.4 chimeric reads/Mb for *E. coli* and *B. subtilis*, respectively. In contrast, reads classified as potentially chimeric contained 3,137 ± 216 and 3,291 ± 222 chimeric reads/Mb, while raw reads initially contained 727 ± 125 (17%) and 410 ± 27 (13%) chimeric reads/Mb, indicating that a single cycle of cross-reference mapping identifies most chimeras. However, discarding such chimeras may also result in excessive loss of genetic information. Indeed, while genome coverage for clean reads was comparable to that of unprocessed reads at 77 ± 13% for *E. coli* and 80 ± 25% for *B. subtilis*, potentially chimeric reads also covered 69 ± 12% and 65 ± 22% of the genome, respectively (Fig. 2c,d). Hence, potentially chimeric reads contain significant levels of genetic information that may eventually fill gaps and enable assembly of long contigs. To recapture this information, potential chimeras were then split based on alignment and reclassified by cross-reference mapping until potentially chimeric reads become undetectable after multiple cycles. Typically, more than 95% of total reads were classified as clean within three cycles, with chimeras significantly decreasing to 0.2% and 0.02% of total *E. coli* and *B. subtilis* clean reads (Table 1). Similarly, some reads classified as unmapped at the end of cross-reference mapping are, indeed, fully mappable to the reference genome (Table 1), but underrepresented in raw SAGs. Although covering only a small fraction of the genome (Fig. 2c,d), these reads were used to close gaps between contigs as described below. Strikingly, not only did multiple cycles of classification reduce chimeric reads from 727 ± 125 reads/Mb to 9 ± 5 reads/Mb in *E. coli*, and from 539 ± 28 reads/Mb to 1.0 ± 0.5 reads/Mb in *B. subtilis*, the number of chimeric reads in cleaned SAGs was lower than in reads obtained from bulk *E. coli* (184 reads/Mb) and *B. subtilis* (130 reads/Mb) genomic DNA (Fig. 2e). This indicates that cross-reference processing potentially removes chimeric artifacts not only from MDA, but also from library preparation using Nextera XT (see Methods).

**Table 1:** Distribution of sequence reads after cycles of SAG cross-reference mapping and chimera splitting

Mapping result to reference genome	Raw SAG	After 1^st^ cycle of classification			After full classification		
		Clean	Potential chimera	Others (unmapped)	Clean	Potential chimera	Others (unmapped)
*E. coli*							
Fully mapped	1,735 ± 258	1,498 ± 232	237 ± 47	2.7 ± 1.1	2,102 ± 290	—	17 ± 15
Chimeric	360 ± 57	2.6 ± 1.0	355 ± 57	0.02 ± 0.02	4.1 ± 2.2	—	1.2 ± 0.9
Unmapped	5.4 ± 6.1	4.6 ± 5.4	1.4 ± 1.6	0.3 ± 0.1	20 ± 16	—	88 ± 40
*B. subtilis*							
Fully mapped	1,884 ± 208	1,700 ± 193	183 ± 23	1.1 ± 0.7	2,096 ± 231	—	14 ± 16
Chimeric	219 ± 28	0.4 ± 0.2	219 ± 28	0.02 ± 0.02	0.5 ± 0.2	—	0.7 ± 0.8
Unmapped	0.2 ± 0.2	0.002 ± 0.003	0.03 ± 0.02	0.2 ± 0.2	0.4 ± 0.2	—	66 ± 8

Data are mean (±s.d.) of 12 SAG data sets (2 M reads) per species and represent read numbers (×10^3^ reads).

### Assembly of cleaned SAG reads into composite single-cell genomes

Misassembled contigs constructed by *de novo* assembly (Fig. 2f) were significantly fewer in cleaned *E. coli* (10 ± 3 contigs) and *B. subtilis* (1.3 ± 0.7 contigs) reads than in raw reads (59 ± 9 contigs and 28 ± 15 contigs, respectively), suggesting that cleanup by cross-reference mapping improves the quality of *de novo* assembly.

**Figure 3:**
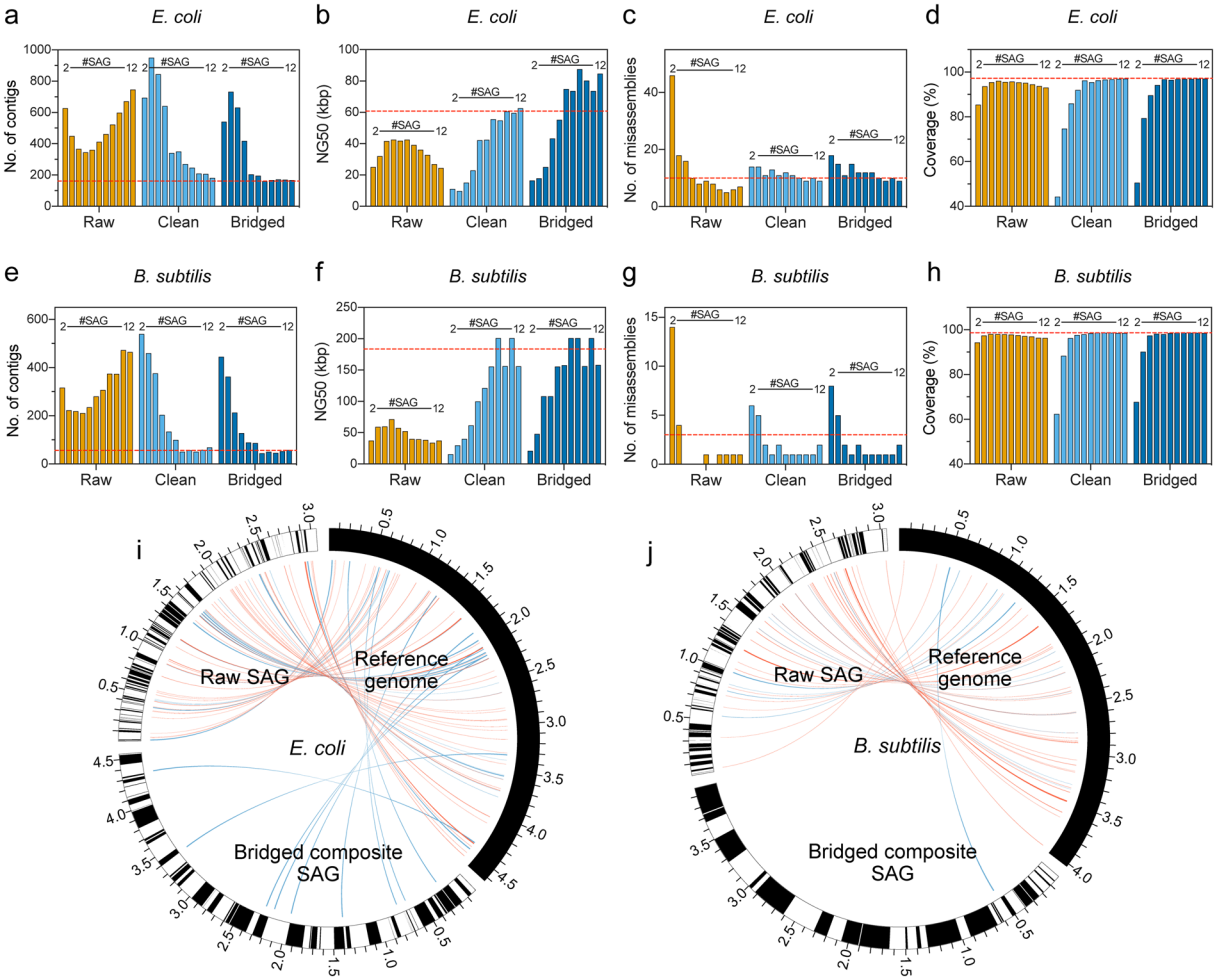
*De novo* co-assembly of multiple SAGs into near-complete composite single-cell genomes. Co-assembly of 2–12 clean (**a**–**d**) *E. coli* and (**e**–**h**) *B. subtilis* SAGs. After *de novo* co-assembly, the (**a** and **e**) number of contigs, (**b** and **f**) NG50, (**c** and **g**) number of misassembled contigs, and (**d** and **h**) coverage were calculated for each composite single-cell genome, and compared with those of a genome obtained from 10 ng bulk DNA (red dashed lines). (**i** and **j**) Circos plots comparing contig size and sequence accuracy between raw SAG contigs and bridged composite SAG contigs. Contigs are drawn as black and white bins with total contig length indicated in Mb. Points of relocation and inversion relative to reference genomes are shown in blue and red ribbons, respectively.

By co-assembling 2–12 SAG data sets into composite genomes (Fig. 3a–h), we found that integration of five raw SAGs best improved the number of contigs (Fig. 3a,e), NG50 (Fig. 3b,f), number of misassemblies (Fig. 3c,g), and genome coverage (Fig. 3d,h). In particular, the co-assembling of multiple SAGs prevents apparent misassemblies regardless of the cleaning and bridging processes. However, integration of >5 raw SAGs degraded assembly qualities such as the contig number, NG50, and coverage due to the accumulation of incorrect sequences such as chimeras. On the other hand, slight degradation in assembly quality was observed when less than 5 SAGs were cleaned and integrated, presumably because about 20% of randomly dispersed genomic sequences were lost, and because reads that would have otherwise been considered as consensus were instead discarded as unmapped. However, cleaning and integration of ≥6 SAGs, generated clean composite SAG contigs with quality equivalent to those of contigs obtained from bulk genomic DNA. Of note, cleanup had a profound effect on assembly quality in *B. subtilis*, which was better covered than *E. coli* due to low GC %. Taken together, these results indicate that chimera removal from and integration of a sufficient number of SAG data sets may yield clean and large contigs. However, if too few SAGs are available or if SAGs have low coverage, many correct sequences may be flagged as nonconsensus and eliminated during assembly. To address this potential issue, we investigated the possibility of using contigs from raw SAGs to bridge clean composite SAG contigs. Notably, assembly quality equivalent to that obtained from bulk genomic DNA was achieved with eight fewer SAGs when bridging and gap-filling from raw contigs was performed (Fig. 3a–h).

By integrating all 12 SAGs, a composite SAG was obtained from *E. coli* with 167 contigs, NG50 of 72.0 kb, 9 misassemblies, and coverage 97.1%. Similarly, a composite *B. subtilis* SAG was obtained with 58 contigs, NG50 of 158.1 kb, 2 misassemblies and coverage 98.6%. Circos plots comparing bridged, composite SAG contigs with reference genomes (Fig. 3i,j) indicate that errors such as relocations and especially inversions are significantly and dramatically reduced by ccSAG. Moreover, bridged, composite SAG contigs were long and contiguous, while ungrouped raw SAG contigs were short and fragmented. The improvement in assembly quality was also confirmed on Bandage assembly graphs^19^, shown in Supplementary Fig. S1. In these graphs, integration of short, fragmented, and contaminated raw SAGs resulted in a complex and tangled assembly with many similarly short contigs. However, ccSAG cleanup and integration of multiple SAGs resulted in a composite SAG similar to that obtained from bulk genomic DNA. Collectively, the data indicate that the ccSAG workflow, which includes cross-reference mapping, chimera splitting, integration of cleaned SAGs, *de novo* co-assembly, and contig bridging, generates a composite single-cell genome with high accuracy and coverage.

### Comparison of ccSAG and jackknifing

We then compared the performance of ccSAG against jackknifing^17,20^, which was reported to improve assembly quality when three or more SAGs are used to identify chimeras over 4–6 cleaning cycles. By co-assembling 12 SAGs each of *E. coli* and *B. subtilis* without cleaning (Table 2), the contig qualities were improved compared to those of the ungrouped raw SAG contigs. The jackknifing cleaning yielded high genome coverage (>96%) and large contigs (>170 kb), but the total contig length became larger than the actual genome size of both species due to incomplete coverage. In contrast, ccSAG generated fewer contigs with long NG50, high coverage (>97%), and with total length close to the actual genome size (4.6 Mb for *E. coli* and 4.0 Mb for *B. subtilis*). As is clear from Table 2 and Supplementary Fig. S1, the co-assembly of cleaned reads by ccSAG clearly facilitated a reduction in short contigs while maintaining the total contig size and genome coverage. Moreover, the contig bridging process improved contig length by integrating all the sequences necessary for complete genomes. However, as shown in the *B. subtilis* data, ccSAG may have little to no effect in reducing apparent misassemblies in the case of SAG data with fewer inherent errors. Overall, this result indicates that ccSAG results in superior genome assembly quality compared with conventional jackknifing. We attribute this difference in performance to the ability of ccSAG to effectively remove spurious sequences from complex SAG sequence data, and assemble long and clean contigs after recycling potentially chimeric reads and gap-filling with single-copy reads. In particular, ccSAG can provide cleaned composite contigs with much less computational time (<18 hours) compared to jackknifing (>96 hours).

**Table 2:** Comparison of sequence cleanup and co-assembly by ccSAG and jackknifing.

*Index*	Ungrouped raw SAG (average)	Co-assembled raw SAG	Co-assembled clean SAG		
			Jackknifing	ccSAG	
				Clean	Bridged
*E. coli*					
Contigs ≥ 0.5 kb	717	747	185	182	167
Contigs ≥ 2 kb	253	248	119	127	115
Largest contig (kb)	79	112	185	180	180
Total length (Mb)	3.48	4.49	4.65	4.63	4.63
NG50 (kb)	11.7	23.8	62.1	62.6	84.7
Misassemblies	59	7	10	9	9
Coverage (%)	63.60	93.15	97.00	97.03	97.05
Computational time (hour)	—	—	97	14.0	17.5
*B. subtilis*					
Contigs ≥ 0.5 kb	388	465	99	69	58
Contigs ≥ 2 kb	150	181	40	50	47
Largest contig (kb)	140	123	303	356	356
Total length (Mb)	3.38	3.94	4.02	3.97	3.97
NG50 (kb)	36.1	35.4	155.8	155.9	158.1
Misassemblies	28	1	1	2	2
Coverage (%)	83.52	96.36	98.65	98.57	98.58
Computational time (hour)	—	—	110	13.5	16.5

*E. coli* and *B. subtilis* SAGs (n = 12) were cleaned and co-assembled by ccSAG or by jackknifing with 6 cleanup cycles. The computational times were estimated with 24 CPU cores.

### Composite single-cell genomes from uncultured mouse gut microbes

**Figure 4:**
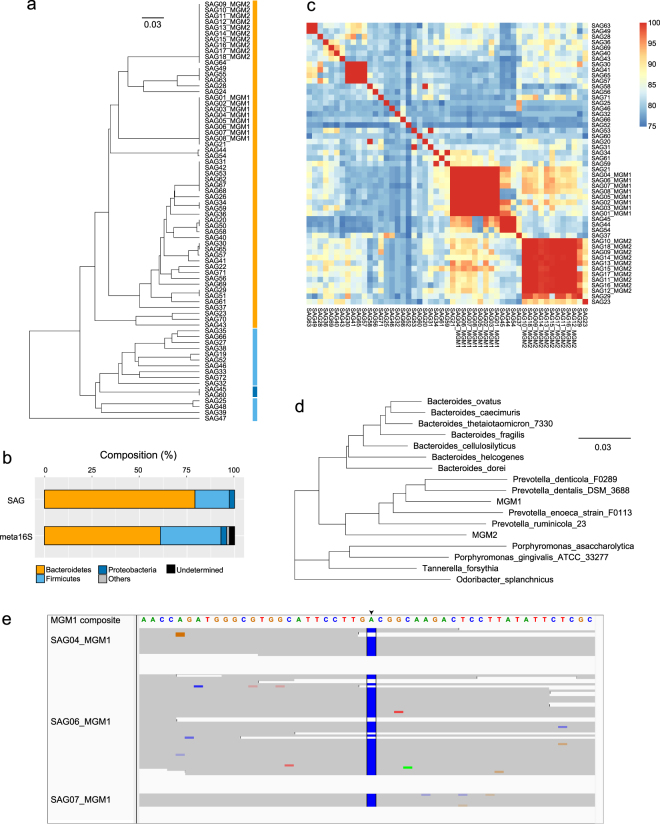
Single-cell sequencing of mouse gut microbes by ccSAG. SAGs from mouse gut microbes (n = 72) were obtained by single-droplet MDA, sequenced, and processed by ccSAG to obtain composite single-cell genomes. (**a**) Phylogenetic tree based on 16S rDNA V3-V4. Phyla are highlighted in different colors. (**b**) Distribution of gut microbial phyla as determined from SAGs and metagenomic 16S rDNA. (**c**) Mean pairwise genomic similarity, as measured by BLAST. Strongly contaminated (>10%) samples or samples with no alignments were excluded from this analysis. (**d**) Phylogenetic tree of MGM1, MGM2, and mammal-associated *Bacteroidales* based on full length 16S rRNA. (**e**) Sequence mapping of cleaned reads of a putative polysaccharide lyase gene from single MGM1 cells (SAG04, SAG06, and SAG07), with the composite single-cell genome as reference. The composite genome is color-coded by base, and SNPs (A to C) in each read in SAG04, SAG06, and SAG07 are highlighted in corresponding base colors.

Using single-cell whole-genome amplification in single droplets^12^, 72 SAGs from mouse gut microbes were simultaneously acquired from individual droplets randomly collected in a single experiment. *De novo* assembled contigs from raw SAGs had median genome completeness 67.2% and median contamination 1.98% (Supplementary Data S1). Based on >96% similarity in 16S rDNA V3-V4 (Fig. 4a), 80% of SAGs were assigned to *Bacteroidetes*, while 17% were assigned to *Firmicutes* (Fig. 4b). The phylogenetic distribution of these SAGs was slightly different from that of 16S rDNA acquired from a metagenomic sample, and the SAGs cover 34 of 379 operational taxonomic units (9.0%). These differences are attributed to variations in 16S rRNA gene copy number in bacterial genomes.

Notably, the *Bacteroidetes* strains MGM1 and MGM2 were represented by at least eight SAGs each, with 16S rRNA identity ≥99% (Fig. 4a) and ANI ≥ 98% (Fig. 4c). These 16S rRNA sequences were also similar to other *Bacteroidales* 16S rRNA, although nucleotide identity against the nearest known bacterial genomes CP002589 and CP002006 was only 88.8% and 85.0%. Thus, we constructed composite single-cell genomes for MGM1 (n = 8) and MGM2 (n = 10) using ccSAG (Table 3). Raw SAGs had average completeness 76.0% and 53.1%, with average contamination 1.99% and 1.59%, respectively. Integration of raw SAGs into raw composite SAG contigs improved completeness, but also increased potential contamination to 9.47% and 4.37%. Upon removal of chimeras by ccSAG, the potential misassemblies of each SAG were reduced by >70% to 20 and 14, respectively, while maintaining the individual genome coverages of the SAGs (70% and 49%, respectively). By co-assembling these cleaned SAGs, contamination was effectively reduced to <1.44%, with only a slight drop in completeness (>96.7%). Bridging of gaps between contigs generated the longest contigs (N50 > 21 kb) with high completeness (>96.7%) and lowest contamination (<1.25%). However, there was some risk of degradation in contig size and number after chimera removal in MGM2 (Table 3), because some SAG data were incomplete and may have amplified the error in chimera identification. Nevertheless, contig bridging rescued correct sequences that may have otherwise been lost, and were then integrated into cleaned data. These results indicate that chimera removal and contig bridging in ccSAG improve *de novo* assembly to a quality consistent with that obtained from lab-cultured cells.

**Table 3:** Comparison of *de novo* co-assembled genomes obtained from SAGs of two mouse gut microbes.

Steps	Contigs ≥0.5 kb	Contigs ≥2 kb	N50 (kb)	Total length (Mb)	Completeness (%)	Contamination (%)
*MGM1 (n* = *8)*						
Unprocessed						
Ungrouped raw SAG contig	531	145	19.0	2.15	75.96	1.99
Raw composite SAG contig	433	138	39.4	3.25	99.25	9.47
After processing						
Clean composite SAG contig	288	141	41.1	2.93	96.93	1.12
Bridged composite SAG contig	225	108	78.5	2.95	97.68	1.12
*MGM2 (n* = *10)*						
Unprocessed						
Ungrouped raw SAG contig	439	135	12.5	1.53	53.07	1.59
Raw composite SAG contig	362	199	20.2	3.02	98.38	4.37
After processing						
Clean composite SAG contig	466	232	15.4	2.83	96.69	1.44
Bridged composite SAG contig	329	198	21.1	2.88	96.69	1.25

The genomic features of MGM1 and MGM2 based on composite single-cell genomes are listed in Table 4. The estimated genome size, GC %, and number of coding sequences are similar between both strains. Importantly, the presence of essential features and highly conserved genes suggest that both genomes are nearly complete. For instance, each genome contains a set of aminoacyl-tRNA synthetases, at least one tRNA for each amino acid, and full-length 5S, 16S, and 23S rRNA genes in contigs of all sizes. MGM1 and MGM2 also appear to be related, based on full length 16S rRNA (85.3% identity, Fig. 4d) and comparison of gene functions to other *Bacteroidales* genomes in GenBank (Supplementary Fig. S2). However, some pathways intact in one species were completely absent in the other. For example, a biosynthetic pathway for cobalamin, a vitamin known to be synthesized by gut microbes^21^, was found only in MGM1. Remarkably, MGM2 and other *Prevotella sp*. discovered from the oral cavity or rumen (CP002589, CP013195, and CP002006) did not code for the complete pathway. Therefore, we believe that MGM1 and MGM2, probably *Prevotella sp*., play different metabolic roles in the mouse gut microbiota.

**Table 4:** Features of the composite single-cell genomes of *Prevotella sp*. MGM1 and MGM2.

Feature	MGM1	MGM2
Total length (Mb)	2.95	2.88
GC content (%)	47.08	46.88
Contigs ≥ 0.5 kb	225	329
Contigs ≥ 2 kb	108	198
N50 (kb)	78.5	21.1
Largest contig (kb)	222.3	149.5
Genome completeness (%)	97.68	96.69
Estimated genome size (Mb)	2.99	2.96
Coding sequences	2,447	2,543
tRNAs	38	36
Copies of rRNA operon	1	1

### Sequence heterogeneity in single strains

By comparing coding sequences between individual and composite SAGs, distinct SNPs were detected in 6 sites from MGM1, 2 sites from MGM2, and 2 sites from *E. coli*. For example, the same SNP (A to C) in a polysaccharide lyase gene occurs at the same site in multiple MGM1 cells (Fig. 4e and Supplementary Fig. S3a), and was above the sequence error frequency due to amplification and sequencing artifacts as described in the methods. These detected SNPs were also confirmed by Sanger sequencing of amplicons (Supplementary Fig. S3b). This SNP site clearly shows two types of nucleotide variations (Fig. 4e) that result in a change in amino acid (Asp to Ala). Therefore, even though cells within the same uncultured strain of MGM1 or MGM2 were considered identical based on 16S rRNA and ANI (Fig. 4a,c), genetic, functional subtypes appear to be present within the respective populations.

## Discussion

Although direct co-assembly of several raw SAGs may increase apparent completeness, chimeric and other contaminant sequences are also accumulated (Figs 2 and 3; Table 3), resulting in degraded assembly quality (Fig. 3). Thus, precleaning and integration of an optimal number of SAGs are critical for obtaining a near-complete composite genome with quality equivalent to those obtained from bulk DNA. In ccSAG, reads from identical microbe strains are first grouped, and then compared to each other to identify nontarget or potentially chimeric reads. This approach is particularly useful for uncultured microorganisms, for which there is no reference sequence data that can be mapped to identify chimeric and other contaminant sequences. Subsequently, cleaned SAG reads are combined to compensate for lack of genome coverage in individual SAGs, noting that reads from single cells of the same strain should cover different portions of genome, since amplification bias and chimeras from MDA occur randomly^1,6,9^. ccSAG also outperforms conventional jackknifing in read cleaning, assembly quality, and computational time. In addition, the former is based on simple read classification prior to read assembly and is thus less computationally demanding than the latter, which requires multiple cycles of SAG assembly and chimera identification.

A certain number of SAGs from taxonomically identical cells is required to ensure sequence representation and overlap. However, the completeness of a SAG is only 40–55% in general, and success rates from environmental samples tend to be <10%^5^. Therefore, in conventional approaches, a large number of single cells are first isolated by fluorescence-activated cell sorting^5,22,23^, whole-genome amplified, and screened for productive reactions. Moreover, these approaches also require stringent workflows to minimize contamination and amplification bias^5,10,11,24–26^. In this light, we recently developed single-droplet MDA, a technique that enables massively parallel single-cell genomics by increasing sample preparation efficiency^12^. In this approach, contaminant reads are effectively reduced to less than 1% due to picoscale reactions instead of tube-scale reactions. In addition, completeness was improved to approximately 50–80%, even from uncultured environmental microbes. Accordingly, this technique is quite compatible for downstream processing by ccSAG.

In conventional SAG co-assembly, SNPs in single cells may disappear from the resulting composite single-cell genome, which is essentially a consensus sequence for the population. By combining single-droplet MDA and ccSAG, long contigs that cover coding sequences are obtained, and can be surveyed for SNPs within the same strain, using the composite genome as internal reference. SNPs were observed in a specific gene in multiple cells of the uncultured microbes, even though cells within this group were considered to be identical based on strict similarity of 16S rRNA and high ANI. This result indicates the presence of heterogenetic subtypes related to polysaccharide utilization within the same gut microbe species. This analysis also highlights the suitability of ccSAG to study genetic heterogeneity in single microbial cells from environmental samples, as well as its ability to minimize contig number without producing erroroneous sequences and generate high-quality genomes.

Using single-droplet MDA, 72 SAGs were acquired from the mouse gut microbiome, covering 9% of operational taxonomic units detected from metagenomic 16S rRNA analysis. Of these SAGs, strains MGM1 and MGM2 were represented in sufficient numbers for analysis by ccSAG. We anticipate that the number of SAG groups suitable for ccSAG analysis would increase with the number of SAGs acquired. Thus, integration of a DNA barcoding scheme with single-droplet MDA and ccSAG may yield composite single-cell genomes from uncultured microbes in massively parallel fashion^12^. In addition, combining short-read sequencing and recently developed long-read sequencing technologies, such as nanopore sequencing, may further improve assembly quality by ccSAG and yield long contigs with high accuracy.

In summary, we have developed ccSAG, a tool to assemble high-coverage and accurate composite single-cell genomes from multiple single-cell sequence data. The ability to obtain composite single-cell genomes with quality comparable to those obtained from bulk genomic DNA provides new opportunities to investigate microorganisms without the need to cultivate or interpret complex metagenomic data. The integration of this workflow with droplet-based single-cell sequencing will enable high-resolution comparative genomics of uncultured microbes at single-cell levels, as well as genetic and functional investigation of microbial dark matters.

## Methods

### Cell line sample preparation

We obtained 12 SAG data each for *E. coli* K12 (ATCC 10798) and *B. subtilis* (ATCC 6633) from Hosokawa *et al*.^12^. In the original paper, these cells were acquired from the ATCC. *E. coli* K12 was cultured in Luria-Bertani (LB) medium (1.0% Bacto-tryptone, 0.5% yeast extract, 1.0% NaCl, pH 7.0) *B. subtilis* was cultured in Brain Heart Infusion Broth (ATCC medium 44, Thermo Fisher Scientific, San Jose, CA, USA). The collected cells were washed three times with UV-treated Phosphate-Buffered Saline (−) (PBS, Thermo Fisher Scientific) and subjected to single-droplet MDA and sequencing.

### Preparation of mouse gut microbiota

Feces was collected from a male 7-week-old ICR mouse (Tokyo Laboratory Animals Science Co., Ltd., Tokyo, Japan) and homogenized in PBS. The supernatant was recovered by centrifugation at 2000 × g for 2Sec, and centrifuged at 15000 × g for 3 min. The resulting cell pellet was washed twice with PBS, and finally resuspended in PBS.

### Single-droplet MDA

A microfluidic droplet generator and an MDA reaction device were fabricated and used for single-droplet MDA according to previous reports^12^. Prior to analysis, cell suspensions were adjusted to 0.1 cells/droplet to prevent encapsulation of multiple cells in a single droplet. Using the droplet generator, single microbial cells were encapsulated in lysis buffer D2 (QIAGEN, Hilden, Germany), and lysed at 65 °C for 10 min. Cell lysates were then injected into a droplet fusion device and mixed with droplets of MDA reaction mix (REPLI-g Single Cell Kit, QIAGEN) supplemented with Tween-20 and EvaGreen. After collection in PCR tubes, the droplets were incubated at 30 °C for 2 h and at 65 °C for 3 min. For single-cell sequencing, droplets that became fluorescent were individually picked and transferred by micropipette under an open clean bench (KOACH 500-F, KOKEN LTD., Tokyo, Japan) into fresh MDA reaction mix. After 2 h at 30 °C, the enzyme was inactivated at 65 °C for 3 min.

### 16S rDNA sequencing

To confirm amplification from single cells, 16S rRNA gene fragments V3–V4 were amplified and sequenced by sanger sequencing from SAGs obtained by single-droplet MDA. To compare the phylogenetic distribution, 16S rRNA fragments (V3-V4) were also amplified from a metagenomic sample of gut microbiota and sequenced by MiSeq (Illumina, San Diego, CA, USA). Paired-end reads were connected, trimmed, and clustered by UPARSE^27^ into operational taxonomic units at 97% identity. Taxonomy was determined in RDP classifier^28^.

### Library preparation and whole-genome sequencing

Illumina libraries for single-cell sequencing were prepared from products of single-droplet MDA using Nextera XT DNA sample prep kit (Illumina) with Nextera XT Index Kit. Libraries were then sequenced on an Illumina MiSeq system at 2 × 300 paired-end reads.

### Quality control of SAG reads and construction of cross-reference contigs (step 1 in ccSAG)

SAGs were first grouped based on 16S rRNA similarity ≥99% and ANI > 95%. Nucleotide identity was estimated by pairwise BLAST between full-length raw SAG contigs, and was calculated over ≥500 bp. Grouped SAG reads were then pre-filtered using FASTX-toolkit (http://hannonlab.cshl.edu/fastx_toolkit/) and PRINSEQ^29^ to remove low-quality reads (≥50% of bases with quality scores < 25), trim the 3′-end of reads with low-quality bases (quality score < 20), remove short reads (<20 bp) and reads with 1% of bases unidentified, and discard unpaired reads after such prefiltration. Subsequently, contigs were individually assembled *de novo* from raw SAG reads using SPAdes-3.9.0 with options–careful–disable-rr–sc^16^. Finally, raw SAG contigs ≥500 bp were collected for cross-reference mapping.

### Removal of chimeric reads by cross-reference mapping (step 2 in ccSAG)

Quality-controlled reads from one SAG were mapped by BWA to multiple raw contigs constructed from other SAGs in the same group^30^. A read was considered clean if complete alignment to reference contigs was equally or more frequent than partial alignment (soft clipping), but considered potentially chimeric if partial alignment was more frequent than complete alignment. Potential chimeras were then split into aligned and unaligned fragments, which were then remapped to multiple raw contigs and reclassified as described. Finally, fully unaligned reads and fragmented chimeras shorter than 20 bp were discarded as unmapped. Cycles of cross-reference mapping and chimera splitting were repeated until partially aligned, potentially chimeric reads were undetectable.

### Co-assembly of clean SAGs and contig extension (step 3 in ccSAG)

Clean reads from each SAG were co-assembled *de novo* using SPAdes into clean composite SAG contigs. Similarly, raw SAG reads were co-assembled *de novo* into raw composite SAG contigs. Gaps between clean composite contigs were filled by BLAST mapping against raw composite contigs. Briefly, potentially usable raw composite contigs were identified by ≥99% identity to clean composite contigs over ≥250 bp. Such raw composite contigs were then collected into a database, against which clean composite contigs were mapped by BLAST and gap-filled based on the resulting alignments, thereby generating bridged composite SAG contigs, which essentially comprise the composite single-cell genome.

### Analysis of SAG assembly

Assembly quality was evaluated by QUAST^31^. For the analysis of cell lines, all sequence data were mapped to the NCBI reference genome of NC_00913 (*E. coli* substrain MG1655) with f-plasmid and lamda phage sequence or NCBI reference genome of NC_014479 (*Bacillus subtilis* subsp. *spizizenii* str. W23). For the analysis of uncultured cell genomes obtained by this study, bridged composite SAG contigs were used as references to identify potential misassemblies and determine the genome fraction of each SAG. Completeness and contamination were evaluated by CheckM^14^. Taxonomy was assigned in AMPHORA2^32^ or by BLAST search of 16S rDNA sequences in RNAmmer^33^. Gene pathway analysis was performed in KAAS^34^ and MAPLE^35^, while assembly graphs were generated in Bandage^19^. For the analysis of SNPs, each single-cell-amplified genome was mapped onto the coding sequences of the bridged composite SAG contigs, and then the nucleotides were screened for sites with a coverage depth of at least 5 reads where 99.9% of reads did not match the reference and showed homogeneous bases. After that, nucleotide sites that contained both multiple matched SAGs and unmatched SAGs in same strains were identified as SNPs.

### Ethics approval

All protocols of animal studies were approved by the Committee for Animal Experimentation of the School of Science and Engineering at Waseda University (No. 2016-A137) and in accordance with the law (No. 105) passed by and notification (No. 6) of the Japanese Government.

### Data availability

SAG data of cultured cell lines (*E. coli* and *B. subtilis*) were obtained from DNA Data Bank of Japan (DDBJ) under the accession number DRA005326^12^. Sequence raw data obtained from mouse gut microbe and assembled genome of MGM1 and MGM2 were deposited in DNA Data Bank of Japan (DDBJ) under the accession number PRJDB6267.

Source code and binaries of ccSAG are freely available at https://github.com/mstkgw/ccSAG. ccSAG is supported on OS X and Linux.

## Electronic supplementary material

Supplementary Information

Supplementary Data S1
