# Supplementary material for: Obtaining high-quality draft genomes from uncultured microbes by cleaning and co-assembly of single-cell amplified genomes
Source: Sci Rep. 2018 Feb 1;8:2059. doi: 10.1038/s41598-018-20384-3 (PMC5794965; doi:10.1038/s41598-018-20384-3)
Supplement: Supplementary file 1 — Supplementary Information [file 41598_2018_20384_MOESM1_ESM.doc]

**Supplementary Information: Obtaining high-quality draft genomes from uncultured microbes by cleaning and co-assembly of single-cell amplified genomes**

Masato Kogawa^1,2†^, Masahito Hosokawa^3,4†^, Yohei Nishikawa^1^, Kazuki Mori^2^, and Haruko Takeyama^1,2,3^*

^†^These authors contributed equally

^1^Department of Life Science and Medical Bioscience, Waseda University, 2-2 Wakamatsu-cho, Shinjuku-ku, Tokyo 162–8480, Japan

^2^Computational Bio Big-Data Open Innovation Laboratory, AIST-Waseda University, 3-4-1 Okubo, Shinjuku-ku, Tokyo 169–0072, Japan

^3^Research Organization for Nano & Life Innovation, Waseda University, 513 Wasedatsurumaki-cho, Shinjuku-ku, Tokyo 162–0041, Japan

^4^PRESTO, Japan Science and Technology Agency (JST), 5-3 Yonban-cho, Chiyoda-ku, Tokyo 102–0075, Japan

Correspondence and requests for materials should be addressed to H.T. (email: haruko-takeyama@waseda.jp)


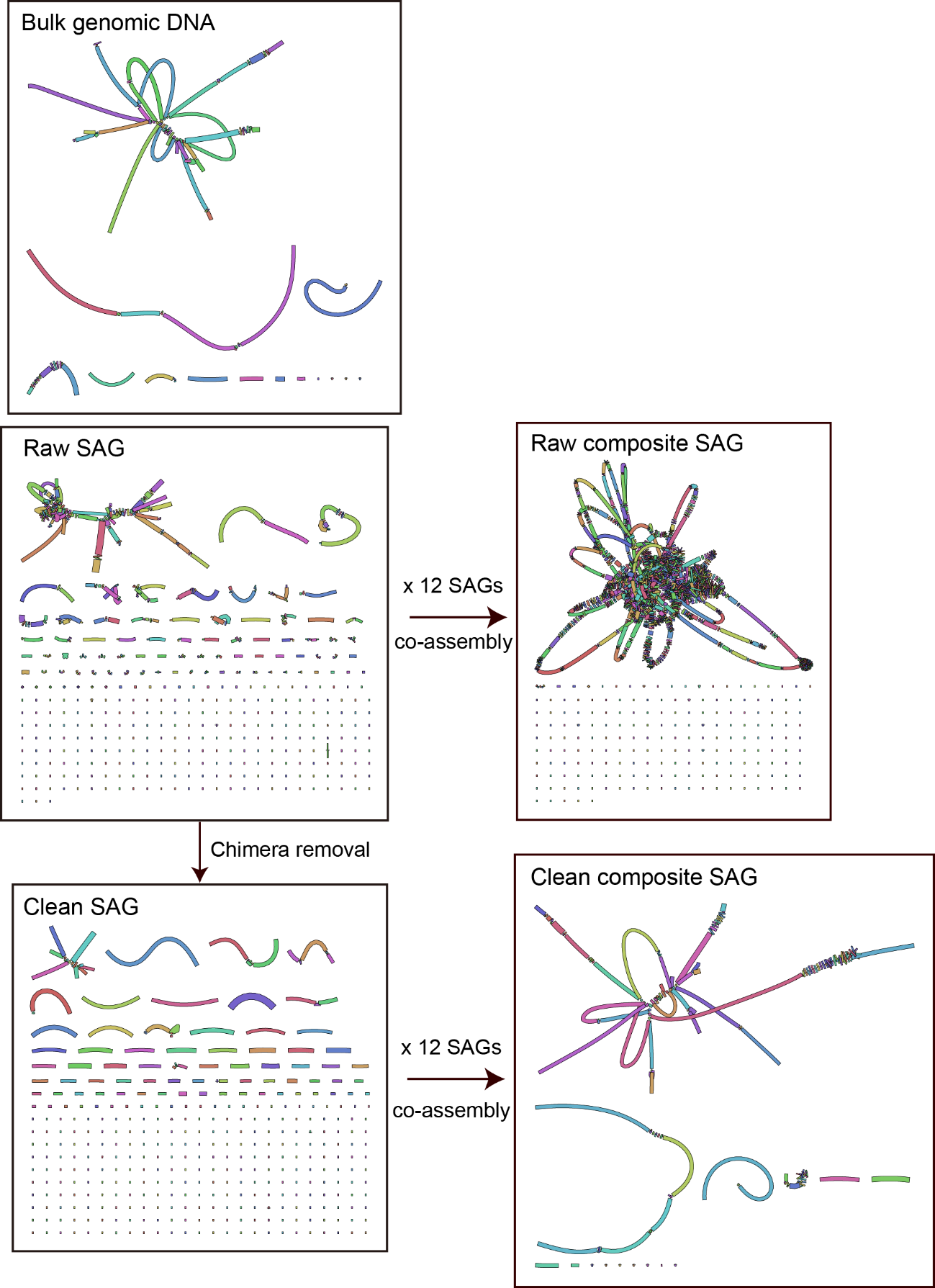
**Fig. S1** Assembly graphs of *B. subtilis* SAG contigs processed by ccSAG. Graphs were produced in Bandage at the same scale.

**Fig. S2** Pathways conserved in mammal-associated *Bacteroidales* genomes, MGM1, and MGM2, with gene frequencies plotted according to the color bar.


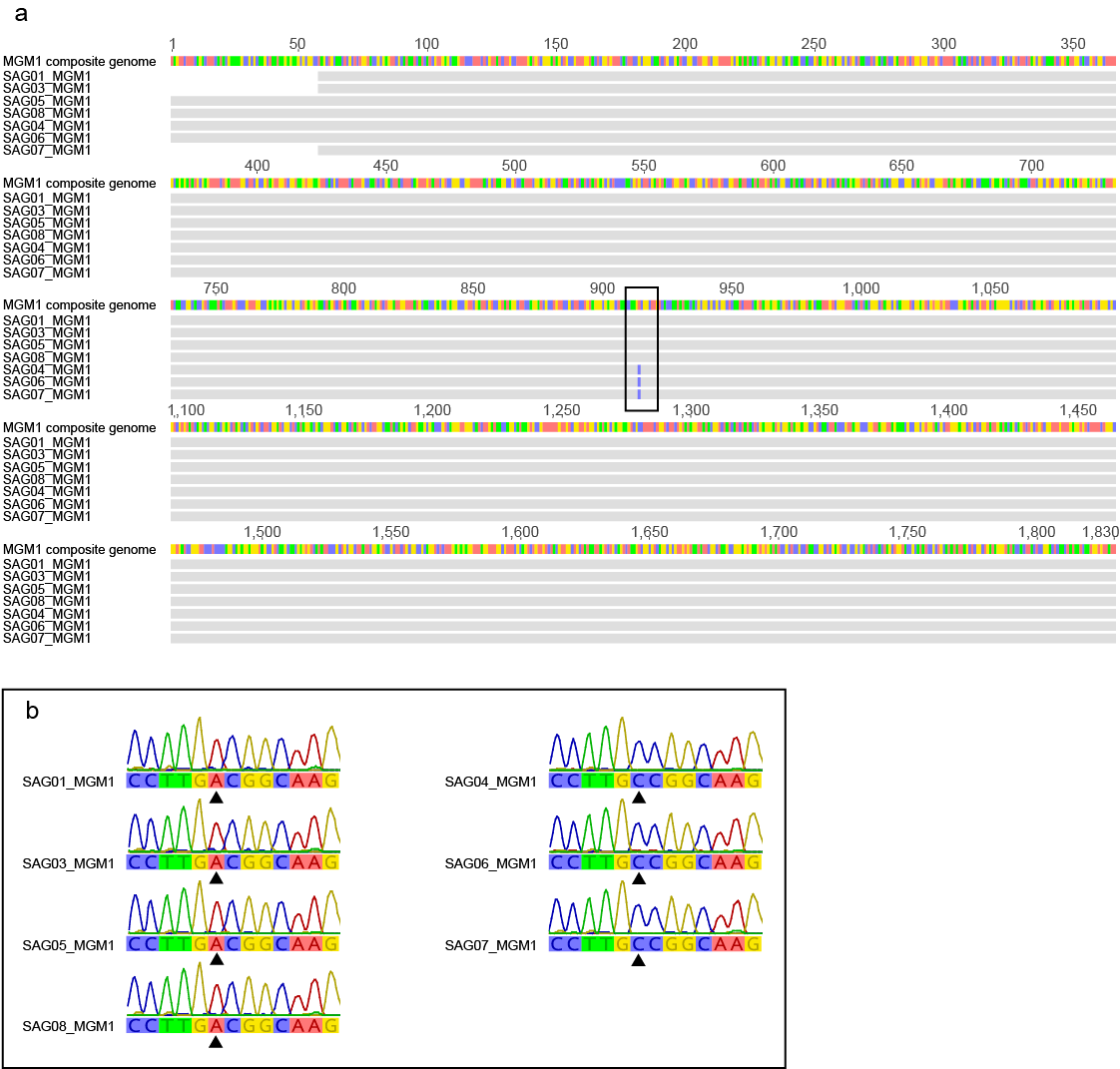


**Fig. S3** Sequences of a putative polysaccharide lyase gene in single MGM1 cells, with the composite single-cell genome (contig no. 199) as reference. Whole contig sequences (a) are aligned to the composite genome. The composite genome is color-coded by base (A: red, T: green, G: yellow, and C: blue), and SNPs in SAG04, SAG06, and SAG07 are highlighted in corresponding base colors (blue). The sequences including the SNPs (base789-base1182) were re-sequenced by Sanger sequencing. The sequencing results of individual SAGs (b) clearly showed the SNPs indicated by arrowhead.
